# The impact of omentoplasty on the reduction of overall complications in liver surgery: an analysis using prediction interval

**DOI:** 10.1097/JS9.0000000000001693

**Published:** 2024-05-22

**Authors:** Jiajun Chen, Jiao Huang, Xinyi Li

**Affiliations:** a Stomatological Hospital of Chongqing Medical University; b Chongqing Key Laboratory of Oral Diseases and Biomedical Sciences; c Chongqing Municipal Key Laboratory of Oral Biomedical Engineering of Higher Education, Chongqing, People’s Republic of China

*Dear Editor,*

We have read with great interest the study conducted by Peng *et al*.^1^, which investigated the efficacy of omentoplasty (OP) in reducing postoperative complications associated with various surgical procedures. Through a comprehensive analysis of 91 studies involving a total of 25 273 patients, the authors convincingly demonstrated that OP was significantly correlated with a decreased risk of overall postoperative complications in gastrointestinal [risk ratio (RR)=0.53] and liver surgery (RR=0.54). Furthermore, OP exhibited significant effectiveness in reducing the incidence of postoperative infections in thoracic (RR=0.38) and liver surgery (RR=0.39). The authors deserve commendation for their timely and rigorously conducted synthetic analysis, addressing a highly relevant clinical question. This study will undoubtedly provide valuable guidelines for surgeons in future clinical practice. However, there are several concerns that necessitate further clarification.

First, in Figure 3 of the manuscript, the descriptions are completely inconsistent with the corresponding forest plots. For instance, Figure 3A actually represents overall complications instead of infectious complications. The authors are required to make subsequent corrections to rectify this error.

Second, as a primary outcome of this meta-analysis, we observed a significant heterogeneity (*I* ^2^=58%) in the analysis of overall complications incidence (Fig. 3A) in liver surgery. Despite conducting sensitivity analysis, the authors were unable to identify the source of this heterogeneity, which could introduce uncertainty into the estimated pooled effect and potentially impact clinical decision-making for this patient population. To further explore the robustness of this finding, we computed a 95% prediction interval (PI) based on the raw data from the meta-analysis to encompass the anticipated range of effects surrounding the summary estimate. By considering both inter-study variability and uncertainty surrounding the summary effect, a PI indicates where we would expect ~95% of analogous future studies’ true effect sizes to lie^2^. If this PI encompasses clinically significant benefits and harms, it implies ambiguity regarding the overall net benefit or harm from forthcoming studies^3^. The PI, which was calculated using R software (version 4.1.2; R Core Team, URL: http://www.R-project.org/), exhibited a PI range for overall complications from 0.18 to 1.62 (Fig. 1). Given that this interval encompasses the null value of 1, it implies substantial uncertainty regarding the pooled effect and underscores the need for cautious interpretation of this finding as well as further high-quality studies to validate this matter. Additionally, we recommend the authors to further investigate potential contributing factors to heterogeneity, such as age, BMI, type of surgery, etc., in order to enhance the credibility of the result and accurately identify the specific liver surgery population that truly requires OP.

**Figure 1:**
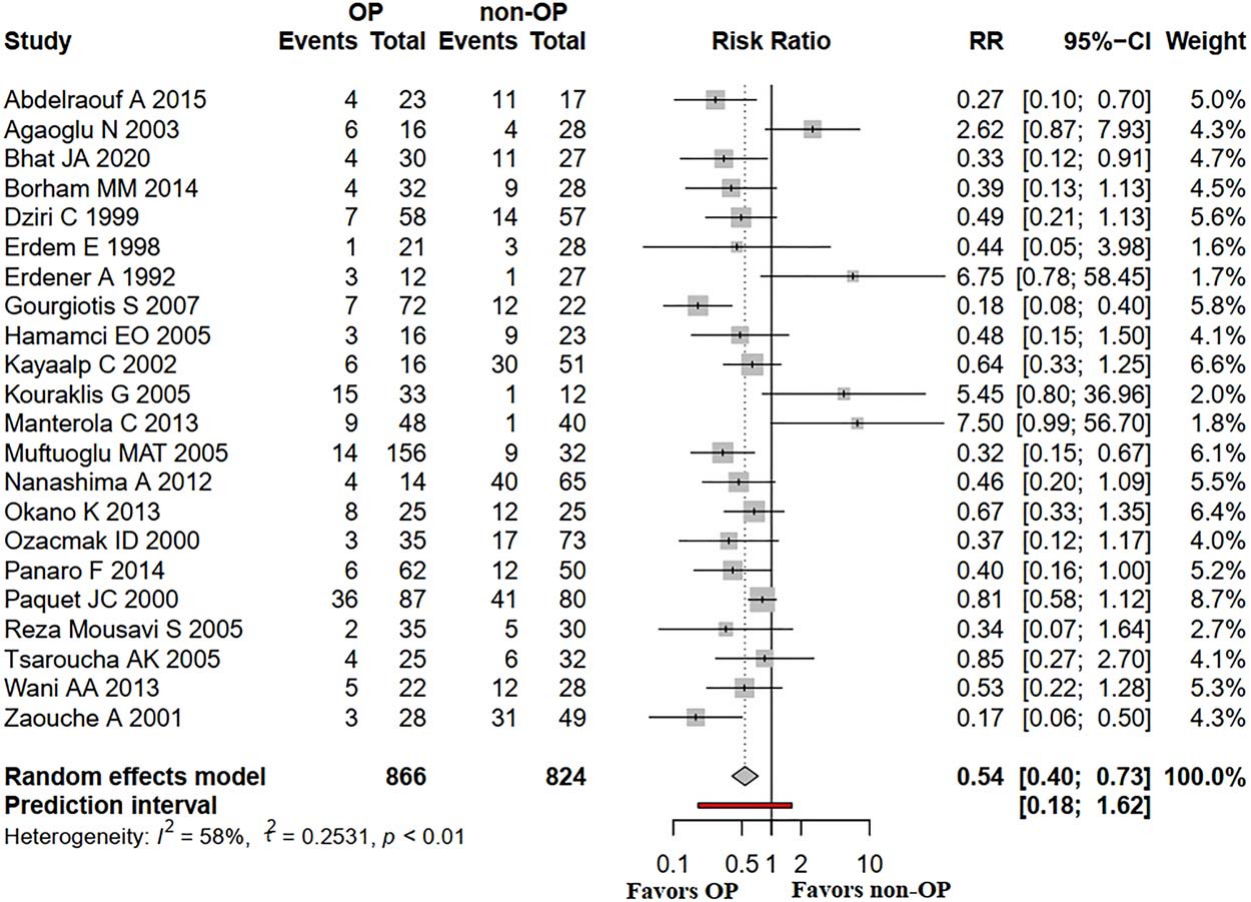
Forest plot displaying pooled risk ratio (RR) with 95% confidence interval (CI) and 95% prediction interval (PI) for the association between omentoplasty (OP) and overall complications incidence in liver surgery.

Overall, we would like to express our appreciation to Peng *et al*.^1^ for their diligent efforts in investigating the role of OP in various surgical procedures. Although further confirmatory evidence is required to substantiate some of their conclusions, this study underscores the potential significance of this approach in ameliorating postoperative complications.

## Ethical approval

Not applicable.

## Sources of funding

This study was supported by the National Natural Science Foundation of China (Nos. 82071115 and 82220108019).

## Author contribution

J.C. and J.H.: original draft conception and writing; X.L.: critical revision of the manuscript. All authors reviewed the manuscript.

## Conflicts of interest disclosure

The authors have no conflicts of interest to declare.

## Research registration unique identifying number (UIN)

1. Name of the registry: none.
2. Unique identifying number or registration ID: none.
3. Hyperlink to your specific registration (must be publicly accessible and will be checked): none.

## Guarantor

Xinyi Li.

## Data availability statement

Not applicable.

## Provenance and peer review

Commentary, internally reviewed.
